# Hybrid Attention-Enhanced Xception and Dynamic Chaotic Whale Optimization for Brain Tumor Diagnosis

**DOI:** 10.3390/bioengineering12070747

**Published:** 2025-07-09

**Authors:** Aliyu Tetengi Ibrahim, Ibrahim Hayatu Hassan, Mohammed Abdullahi, Armand Florentin Donfack Kana, Amina Hassan Abubakar, Mohammed Tukur Mohammed, Lubna A. Gabralla, Mohamad Khoiru Rusydi, Haruna Chiroma

**Affiliations:** 1 Department of Computer Science, Faculty of Physical Sciences, Ahmadu Bello University, Zaria 810006, Nigeria; iatetengi@abu.edu.ng (A.T.I.); ihhassan@abu.edu.ng (I.H.H.); abdullahilwafu@abu.edu.ng (M.A.); donfackkana@abu.edu.ng (A.F.D.K.); ahabubakar@abu.edu.ng (A.H.A.); 2 National Cereals Research Institute, Badeggi 912104, Nigeria; mm.tukur@ncribadeggi.org.ng; 3 Department of Computer Science, Applied College, Princess Nourah bint Abdulrahman University, P.O. Box 84428, Riyadh 11671, Saudi Arabia; lagabralla@pnu.edu.sa; 4 Faculty of Economics and Business, Universitas Brawijaya, Kota Malang 65145, Indonesia; khoiru.r@ub.ac.id; 5 College of Computer Science and Engineering, University of Hafr Batin, Hafar Al Batin 39524, Saudi Arabia

**Keywords:** brain tumor, Figshare, SARTAJ, Br35H, learning rate scheduler, transfer learning, deep convolutional neural network, attention mechanism, progressive image resizing

## Abstract

In medical diagnostics, brain tumor classification remains essential, as accurate and efficient models aid medical professionals in early detection and treatment planning. Deep learning methodologies for brain tumor classification have gained popularity due to their potential to deliver prompt and precise diagnostic results. This article proposes a novel classification technique that integrates the Xception model with a hybrid attention mechanism and progressive image resizing to enhance performance. The methodology is built on a combination of preprocessing techniques, transfer learning architecture reconstruction, and dynamic fine-tuning strategies. To optimize key hyper-parameters, this study employed the Dynamic Chaotic Whale Optimization Algorithm. Additionally, we developed a novel learning rate scheduler that dynamically adjusts the learning rate based on image size at each training phase, improving training efficiency and model adaptability. Batch sizes and layer freezing methods were also adjusted according to image size. We constructed an ensemble approach by preserving models trained on different image sizes and merging their results using weighted averaging, bagging, boosting, stacking, blending, and voting techniques. Our proposed method was evaluated on benchmark datasets achieving remarkable accuracies of 99.67%, 99.09%, and 99.67% compared to the classical algorithms.

## 1. Introduction

The brain is the most vital organ in the human body, which is enclosed by a hard skull. Brain tumors are one of the biggest challenges of modern medicine since they are so complex and carry such a high fatality rate. In brain tumors, there is abnormal growth of brain cells, and any abnormal growth in this confined space can lead to severe complications [1]. Brain tumors are either benign or malignant and are also classified into two major categories: primary and metastatic [2]. Primary brain tumors originate from the brain tissue or its surrounding environment [3], while metastatic tumors occur in other parts of the body and metastasize to the brain via the bloodstream. Primary brain tumors can be benign or malignant, whereas metastatic brain tumors are always malignant [4].

Malignant tumors grow rapidly and can invade adjacent brain tissue, whereas benign tumors usually grow more slowly. Nonetheless, benign tumors also possess serious risks through the compression of surrounding tissues [5]. Approximately 70% of brain tumors are benign, and 30% are malignant [6]. Different primary brain tumors have been identified, including gliomas, astrocytomas, meningiomas, and pituitary tumors [2]. More than 120 different types of brain tumors have been discovered to date, with the most common being meningioma, glioma, and pituitary tumors. Among them, meningioma is a tumor arising from the meninges, covering membranes of the brain and spinal cord, and is hence one of the most frequently encountered primary intracranial tumors [7]. Gliomas, however, arise from glial cells named astrocytes. Astrocytomas, one variety of gliomas, are generally low-grade and indolent. Yet high-grade gliomas are among the most virulent brain tumors. Pituitary tumors occur due to the uncontrolled growth of cells in the pituitary gland. Brain tumors need to be identified early since they can be life threatening [5]. Metastatic brain tumors, the most common form of malignant brain tumors, develop most commonly from cancers of the lung, breasts, or skin (melanoma). To invade the brain, these cancer cells must penetrate the blood–brain barrier (BBB) and acclimatize to the brain-specific microenvironment by secreting cytokines, chemokines, and other mediators to help reorganize the tumor microenvironment (TME) [8].

In contemporary medical practice, imaging modalities are being used more by radiologists as they are accurate and pose fewer hazards to patients. For acquiring medical imaging data, various methods have been employed, such as radiography, MRI, tomography, and echocardiography. Among these, MRI is shown to be more beneficial as it provides high-quality images without radioactive exposure. Being a non-surgical technique, MRI gives radiologists useful insights into medical images that contribute to diagnosing brain abnormalities [9,10]. Radiologists mainly utilize MRI scans to detect brain tumors. But with the exponential increase in medical data, it has become ever more difficult to examine and interpret such large data using traditional techniques [11,12]. As diagnosis accuracy, to a great extent, is reliant on the experience of the radiologist, computer-based diagnostic support is in high demand for simplifying the process [1].

Computer-aided diagnosis (CAD) systems offer an encouraging solution where the early detection of brain tumors can be achieved with minimal human interaction. CAD systems generate diagnostic reports from MRI scans and provide additional guidance to radiologists [13]. The classification of brain tumors in CAD includes two main steps: feature extraction and classification. The integration of machine learning (ML) and deep learning (DL) has enhanced the CAD process considerably in medical imaging [14,15,16]. Traditional machine learning methods are feature extraction, selection, and classification-based. Feature extraction was widely popular in the early artificial intelligence era, and the methods employed were the gray level co-occurrence matrix (GLCM), bag-of-words (BoW), Gabor filters, thresholding, clustering, contour-based, and intensity histograms [17,18]. These methods have the tendency to discard valuable information from the source image [19]. Such conventional ML-based approaches are less suitable for image classification as they heavily depend on manually designed features [20]. What makes them particularly well-suited for complex biomedical applications is that they are able to automatically extract optimized features from raw data without the necessity of any human intervention, improving classification performance by tailoring the feature extraction process to the problem in question [21].

The new breakthrough in deep learning (DL) has resolved the limitations of traditional machine learning (ML) feature extraction using the original image as input. This has put convolutional neural networks (CNNs) into the spotlight, which automatically extract the desired features for image classification using a number of convolutional layers [22,23]. DL is an advanced approach for prediction and classification with strong performance on problems involving the multi-level processing of information, such as detection, classification, and voice recognition [24,25].

However, CNNs work optimally with large number data, which are not always available in the case of medical imaging [26]. Transfer learning is one widely used technique to circumvent this problem. In transfer learning [27,28], a pre-trained model on a large dataset from another but related problem domain is adapted for the target classification task [29]. Through the utilization of this pre-trained knowledge, the model is capable of high accuracy even when it has a limited dataset [30]. Transfer learning also conserves computation, since the model benefits from the pre-trained network’s convolutional weights, with the last dense layer being the only one that needs to be trained [31].

The hyperparameters of a CNN play an important role in determining its performance, as they impact the training process of the network. Identifying the best hyperparameters, for instance, learning rate, neurons and layers, batch size, dropout rate, and regularization strength, can significantly enhance CNN accuracy. Manually determining the best values, however, is not a simple task; it requires expertise and time. To address this, metaheuristic techniques are applied to discover the best hyperparameter configurations so that the performance of CNNs is improved [32,33]. The tunability of hyperparameters is one of the primary issues of contemporary machine and deep learning because differing initial values could profoundly influence performance metrics like accuracy [34]. Some researchers have turned to metaheuristic algorithms for optimizing deep learning hyperparameters. For instance, ref. [35] applied Grey Wolf Optimization (GWO) to CNN hyperparameter optimization in the context of skin cancer classification. Similarly, ref. [36] investigated certain metaheuristic algorithms like Genetic Algorithm (GA), Whale Optimization Algorithm (WOA), Multiverse Optimizer (MVO), Satin Bower Optimization (SBO), and Life Choice-Based Optimization (LCBO) to improve CNN performance in the detection of breast cancer abnormalities. Furthermore, ref. [37] applied Particle Swarm Optimization (PSO) to the hyperparameter optimization of CNN to identify early lung cancer.

WOA has also been applied in optimizing CNN hyperparameters since it can efficiently explore complex search spaces. According to the study of [38], the WOA has traits of simplicity and effectiveness in addressing such optimization issues specifically. Ref. [39] demonstrated its ability to enhance neural network performance through the optimization of connection weights, which also has the potential to optimize CNNs. In response, ref. [40] showcased WOA’s convergent stability in water demand estimation for water resources, reinforcing its applicability in dynamically tuning CNN hyperparameters. However, like most other metaheuristic algorithms, the WOA is plagued by a slow convergence rate. To solve this, chaos theory has been incorporated into the optimization process, enhancing the global convergence rate and overall performance. In the Chaotic Whale Optimization Algorithm (CWOA), various chaotic maps are explored to regulate WOA’s crucial parameters for balancing exploration and exploitation [41]. The traditional CWOA, however, relies on a specific chaotic map while optimizing, which might limit its flexibility in different problem environments. The behavior of chaotic maps may vary depending on the optimization landscape, and therefore, a single map may not always have the optimal search performance. To overcome this limitation, the Dynamic Chaotic Whale Optimization Algorithm (DCWOA) was utilized, introducing a dynamic process where the algorithm oscillates between two different chaotic maps rather than adhering to a static one throughout. This dynamic alternation increases the flexibility of the algorithm and helps it navigate various search spaces more effectively.

In this paper, we present an approach involving the combination of the Xception model and hybrid attention, progressive image resizing, and a Dynamic Chaotic Whale Optimization Algorithm (DCWOA) for hyperparameter optimization. We have a dynamic learning rate scheduler that dynamically adjusts the learning rate during runtime depending on the size of images, with batch sizes and layer-unfreezing schedules that are designed to adaptively optimize training. Several versions of models trained on various image sizes are combined using methods such as weighted averaging, bagging, boosting, stacking, and voting. This integrated method was tested on three datasets and achieved state-of-the-art accuracy, exemplifying its power and applicability in medical diagnostics.

The key contributions of this research can be summarized as follows:

1. Enhanced the Xception model by integrating hybrid attention mechanisms to enhance the ability of feature extraction and emphasize critical regions in the brain tumor images. Additionally, a progressive image resizing strategy was incorporated, enabling models to learn effectively by gradually increasing image sizes during training.
2. Developed a dynamic chaotic variant of the Whale Optimization Algorithm (DCWOA) to optimize key hyperparameters, including the learning rate, the number of neurons, optimizer type, activation function, and dropout rate, ensuring superior model performance and stability.
3. Proposed an adaptive learning rate scheduler that dynamically modifies the learning rate according to the image size, facilitating efficient training across different learning phases and preventing premature convergence.
4. Designed an intelligent batch size selection and layer-unfreezing strategy aligned with image size variations, enhancing training efficiency, convergence speed, and model adaptability to diverse resolutions.
5. Established a comprehensive framework integrating advanced preprocessing techniques, transfer learning-based model reconstruction, fine-tuning, and ensemble strategies, offering a reliable and scalable approach for practical brain tumor diagnosis.

## 2. Related Works

Classifying brain tumors is crucial for early detection and effective treatment planning. Advances in deep learning, especially CNNs, have greatly improved accuracy and automation. Techniques like transfer learning and ensemble methods further enhance performance, with studies confirming the success of CNN-based models applied in MRI-based tumor categorization for high precision and robustness in clinical settings [42,43]. This section reviews recent studies highlighting deep learning’s impact on brain tumor categorization.

A study in [44] proposed the RBP-CNN technique for brain tumor categorization, incorporating gray standard normalization (GSN) for preprocessing, regional binary pattern (RBP) for feature extraction, and a CNN for training, achieving 96% accuracy. Similarly, ref. [45] proposed a multi-level attention network (MANet) for brain tumor identification. The model fuses both cross-channel and spatial attention to augment tumor region discovery without sacrificing temporal dependencies of the semantic features captured from the Xception backbone. MANet produced top accuracies of 94.91% as well as 96.51% on the BraTS and Figshare datasets, respectively.

To improve brain tumor classification, ref. [5] developed two deep learning models, a 23-layer CNN and a fine-tuned VGG16, which effectively identified brain abnormalities and tumor grades with a high accuracy of 97.8%, surpassing state-of-the-art models. However, the refined VGG16 was trained, evaluated, and tested on only one dataset, which undermined its generalizability. Ref. [46] proposed a CNN-based method using the EfficientNetV2B0 architecture (developed by Google Research, Mountain View, USA) with data preprocessing and transfer learning and achieved an impressive classification accuracy of 99.16%, accompanied by recall, high precision, and F1 score. While this shows the efficacy of EfficientNetV2B0, this study could have explored other models of the EfficientNetV2 family for a more comprehensive comparison.

The work of [47] proposed a method whereby features of brain MRI scans from CNNs were distinguished using an SVM, outperforming the CNN using a SoftMax classifier and an accuracy of 95.82%. They found that SoftMax-based CNN classifiers suffered from overfitting due to small dataset sizes. But pre-trained models may have been fine-tuned to address this deficiency. SVMs may also suffer from scalability constraints when handling large datasets and high-dimensional feature sets resulting from CNNs. Similarly, ref. [48] suggested an automated brain tumor identification system utilizing a triple-module architecture based on the integration of known pre-trained deep neural networks, PCA, and a Random Forest classifier. This method, even though it leverages deep learning for feature learning, does not make much use of pre-trained models for end-to-end learning. Moreover, PCA dimension reduction could lead to the loss of significant features, which are critical in medical images to correctly classify the tumors.

A pretrained approach using ResNet152, DenseNet169, VGG19, and MobileNetv3 was suggested in the study of [49] mainly for brain tumor classification, in which MobileNetv3 achieved the highest accuracy of 98.52%. However, validation and testing on the same data result in data leakage, the inflation of performance metrics, and the failure to assess model generalization correctly. A clear division into training, validation, and test datasets would mitigate this issue. Similarly, ref. [50] suggested an ensemble deep learning model by combining a shallow CNN (SCNN) for low-level feature extraction and VGG16 for deeper feature extraction. By feature fusion of the two models to minimize information loss, they achieved 97.77% accuracy. In addition, ref. [51] suggested a privacy-preserving federated learning technique for the categorization of brain tumor based on a customized VGG16 structure to achieve decentralized model training without compromising data security.

The research proposed in [52] utilizes a dilated PDCNN model with several preprocessing techniques and an average ensemble method for categorizing the brain tumors from the MRI scans. The model is completely trained over three datasets, and the accuracy rate is more than 98%, which reflects the success of the model. The average ensemble method, however, may not give good results if models are non-diverse. With weighted averaging or stacking, performance can be improved as strengths can be merged while reducing weaknesses. The research project in [53] foresees an optimization-facilitated hybrid deep learning approach to identify as well as classify brain tumors in the utilized MRI images using the technique exponential deer hunting optimization-based Shepard CNN (ExpDHO-based ShCNN) and also exponential deer hunting optimization-based deep CNN (ExpDHO-based Deep CNN). In their study, ref. [54] utilizes seven new deep learning networks for tumor classification and the detection of tumors in the brain like DenseNet121, InceptionResNetV2, ResNet50V2, Xception, InceptionV3, as well as VGG19 and EfficientNetB7, where maximum accuracy is obtained by InceptionResNetV2 above 97%. However, the use of ensemble methods like stacking, weighted average, or bagging to fuse the predictions belonging to individual trained models can enhance robustness by leveraging the strengths of each model. A combined Quantum Dilated Convolutional Neural Network and Deep Maxout Network (QDCNN-DMN) is proposed by the study of [55] for classifying brain tumors together with increased specificity, accuracy, and sensitivity than traditional techniques. Similarly, ref. [56] presented a WHHO-based deep CNN for the identification of brain tumors using statistical and texture features extracted from MRI images, and optimizing a deep CNN with the WHHO algorithm. The model possessed a best accuracy of 0.816 and sensitivity of 0.974 over other approaches. However, statistical and texture features may lack vital information, whereas feature extraction using deep learning can improve performance by learning important patterns from MRI data directly.

## 3. Materials and Methods

This section offers a summary of the available resources, experimental setup, including the methodologies used in this study. We utilized a combination of datasets, computational tools, and software frameworks to conduct our experiments. The methods employed cover the complete process, from data preprocessing down to model optimization, ensuring a well-structured and efficient approach to achieving our research goals. Every step was carefully designed and systematically executed to guarantee reliability. Special attention was given to selecting the most appropriate techniques, ensuring that the results obtained are meaningful. Python 3.7.10 (Python Software Foundation, Wilmington, DE, USA) was used, and the model was run on the Kaggle cloud platform (Kaggle Inc., San Francisco, CA, USA).

### 3.1. Data Source

Three publicly available datasets, Figshare [57], SARTAJ [58], and Br35H [59], containing grayscale and JPG-formatted MRI scans of the human brain were used in this research. The datasets for all three are representative of the three (3) types of brain tumors: meningioma, glioma, and pituitary tumors. The dataset of Figshare contains 3064 images distributed in 708 meningioma, 930 pituitary, and 1426 glioma cases. Similarly, the SARTAJ dataset holds 3264 images that were labeled as 926 glioma, 937 meningioma, 901 pituitaries, and 500 as no tumor. Br35H is a binary dataset with 3000 images, half of them being 1500 tumorous and 1500 non-tumorous brain MRI scans. These multi-class datasets are a good foundation for the evaluation of classification models used in this work.

### 3.2. Data Partitioning Framework

The datasets employed in this current study were split into three distinct subsets in order to accommodate efficient model creation and testing. Specifically, 80% of each dataset was utilized in training, with the remaining 20% devoted to testing purposes. For even better model performance and prevention against overfitting, the test subset was once again split, with 50% devoted to validation and 50% dedicated to final testing. However, the entire training, validation, and testing procedure was conducted using the TensorFlow 2.11.0 toolkit, taking full advantage of computational resources offered at the Kaggle platform. To prevent data leakage, this study ensured that all data splits were performed on a per-patient basis. This means that images belonging to a single patient were never distributed across training, validation, or test sets. We also used a stratified splitting approach to maintain the class distribution across the different subsets. Furthermore, each dataset was handled independently, and the proposed model was trained separately on each of them to account for differences in class structure and format. This approach ensured that no overlap or contamination occurred between datasets, and patient-level independence was strictly preserved.

### 3.3. Data Preprocessing Pipeline

**Figure 1:**
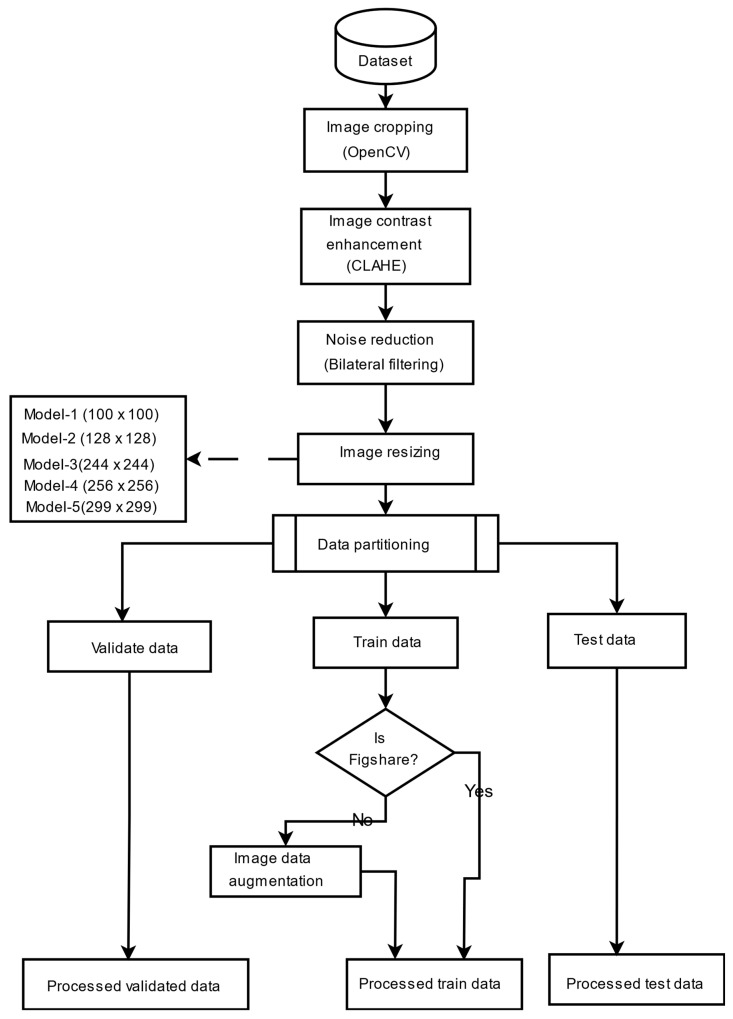
Data preprocessing pipeline.

**Figure 2:**
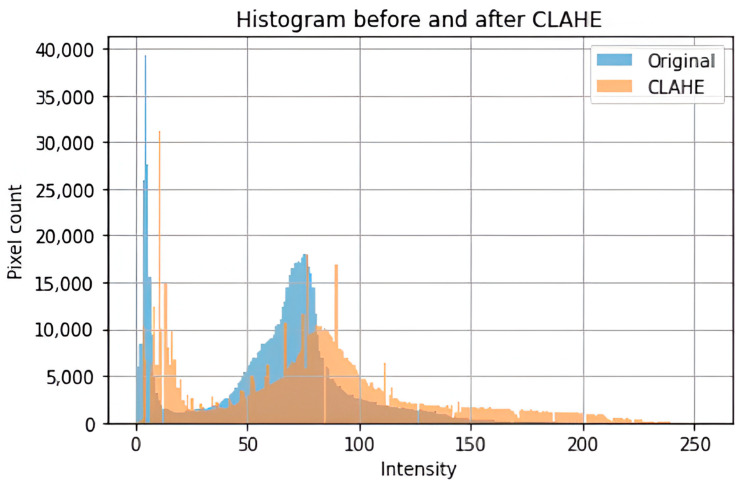
Histogram before and after CLAHE.

To supply the model with a consistent and high-quality input, a complete preprocessing pipeline was implemented to address image size, format, and noise variability in the datasets. The images were cropped to contain just the brain region, which is considered the most significant region to analyze. This was achieved using the popular OpenCV to discover and crop the ultimate top, including bottom, left, as well as right edges of every image. To enhance the saliency of local information and improve feature extraction, contrast-limited adaptive histogram equalization (CLAHE) was employed to boost image contrast. Subsequently, a bilateral filter was applied to reduce noise, particularly from low-contrast areas amplified by CLAHE, without degrading image edges. Progressive image resizing was employed in this research as an integral aspect of the model architecture. This approach employed the step-by-step training of the model using resized images. Each image was resized individually to dimensions of 100 × 100, 128 × 128, 244 × 244, 256 × 256, and 299 × 299 to accommodate the requirements of various training sessions. Resizing was performed as the final step in the preprocessing pipeline to maintain aspect ratio, preserve image features, and avoid loss of salient features. Data augmentation was applied only to two datasets, excluding the Figshare dataset, to tackle the issue of lack of adequately labeled medical image data. No augmentation techniques were applied on Figshare dataset because, during the training phase of the models, we observed the impact of data augmentation on each dataset individually. Our results showed that the application of augmentation techniques (such as rotation, flipping, and scaling) did not lead to any performance improvement on the Figshare dataset. This may be attributed to the dataset’s existing variability and quality, which possibly made further augmentation redundant. However, in contrast, both the SARJAT and Br35H datasets demonstrated improved classification performance when augmentation was applied. This improvement was evident in terms of increased validation accuracy and reduced overfitting, particularly for the smaller and more class-imbalanced Br35H dataset. This technique introduced a huge variety to the training set by adding brightness adjustment, rotation, shearing, horizontal flip, zooming, and width and height shifting. Augmentation was conducted on training subsets after partitioning the datasets to ensure that the CNN model learned invariant features and could differentiate variations. The robustness and model performance improved, and overfitting and underfitting dangers were minimized using this technique. Table 1 provides the data augmentation parameters, and Figure 1 presents the preprocessing flowchart. The preprocessing techniques are also evaluated as shown in Table 2 and Figure 2.

### 3.4. Optimization Algorithm

Optimization methods are instrumental to improving deep learning models by finding optimal hyperparameters that improve performance and avoid overfitting. For this study, we employed a variant of the WOA motivated by the well-known humpback whales’ foraging behavior to improve critical hyperparameters that are used by the Xception model architecture. The algorithm was tailored to adjust parameters such as learning rate, activation function, dropout rate, the first dense layer’s number of neurons, and optimizer. The process takes advantage of the capability of the WOA towards efficiently exploring and exploiting the entire search space so as to obtain optimal hyperparameter selection and better model performance.

#### 3.4.1. Whale Optimization Algorithm

The WOA was introduced in [38]. It is a meta-heuristic optimization method motivated by the hunting habit of humpback whales. The algorithm replicates the bubble net foraging technique, where whales encircle their prey using a combination of circular and spiral bubble patterns to simulate the trapping mechanism. By diving deep and creating spiral-shaped bubbles, the whales effectively contain the prey, preventing its escape. This unique hunting strategy forms the foundation of WOA’s optimization process, as it alternates between chasing prey based on either random or optimal positions and employing a spiral motion to enhance exploitation [41].

#### 3.4.2. Encircling Prey

According to the study of [38], the Humpback whales find and surround targets, and the WOA mimics this by assuming the best current candidate solution represents or is close to the optimal approach. After identifying the best search agent, the other agents update their places toward it. This behavior is illustrated in Equations (1) and (2).

(1) $$\vec{D}=|\vec{C}.\vec{X^{*}}\left(t\right)-\vec{X}\left(t\right)|$$

(2) $$\vec{X}\left(t+1\right)=\vec{X^{*}}\left(t\right)-\vec{A}.\vec{D}$$

Here, t indicates the present iteration, also $\vec{A}$ along with $\vec{C}$ act as coefficient vectors, $X^{*}$ denotes the position vector of the optimal solution found so far, and $\vec{X}$ serves as the position vector. The | | symbol represents the value that is absolute, while dot (.) indicates element-wise multiplication. However, it is important to note that $X^{*}$ is modified in each of the iterations whenever an optimal solution is discovered.

The vectors $\vec{A}$ along with $\vec{C}$ are computed in Equations (3) and (4):

(3) $$\vec{A}=2\vec{a}\;.\;\vec{r}-\vec{a}$$

(4) $$\vec{C}=2\;.\;\vec{r}$$

where $\vec{a}$ is gradually reduced from 2 down to 0 throughout the iterations in the exploration and exploitation stages, and $\vec{r}$ stands as a random vector within the range [0, 1].

#### 3.4.3. Bubble-Net Attacking Method

To mathematically model the bubble-net feeding habit of the humpback whales, the following two strategies are proposed [38]:

1. Shrinking encircling mechanism: This behavior is achieved by gradually decreasing the value of a from 2 to 0 throughout the iterations, as illustrated in Equation (3). The new position of the search agent is determined by selecting the random values for $\vec{A}$ within the range of [−1, 1], which enables the agent to be placed anywhere between its starting position and the position of the current best agent.
2. Spiral updating position: The equation of the spiral, which simulates the helix-shaped movement of humpback whales between the prey’s position and the whale’s position, is expressed in Equation (5).
  (5) $$\vec{X}\left(t+1\right)=\vec{D^{\prime }}\;.\;e^{bl}\;.\cos\left(2\pi l\right)+\vec{X^{*}}(t)$$

$\vec{D^{\prime }}=\left|\vec{X^{*}}\left(t\right)-\vec{X}\left(t\right)\right|$ represents the distance in between the ith whale, along with the prey (current optimal approach or solution). Here, b serves as a constant that determines the structure of the logarithmic spiral, while similarly l is a random number within [−1, 1], and dot (.) signifies element-wise multiplication.

Humpback whales encircle their prey by combining a shrinking circular motion with a spiral path. To represent this behavior, a 50% probability was assigned to choosing either the shrinking encircling mechanism or the spiral model when updating whale positions during optimization using Equation (6).

(6) $$\vec{X}(t+1)=\left\{\begin{array}{cc} \vec{X^{*}}\left(t\right)-\vec{A}\;.\;\vec{D}, & if\;p<0.5\\ \vec{D^{\prime }}\;.\;e^{bl}\;.\;\cos\left(2\pi l\right)+\vec{X^{*}}(t), & if\geq 0.5 \end{array}\right.$$

where *p* serves as a random number in the range [0, 1].

#### 3.4.4. Search for Prey

As stated by [38], the variation in the $\vec{A}$ vector was utilized to facilitate the exploration phase, allowing the questing for prey. By setting $\vec{A}$ to arbitrary values exceeding 1 or falling below −1, search agents were prompted to diverge from a reference whale. The mathematical representation of this phase is formulated as in Equations (7) and (8).

(7) $$\vec{D}=|\vec{C}\;.\;\vec{X_{rand}}-\vec{X}|$$

(8) $$\vec{X}\left(t+1\right)=\vec{X_{rand}}-\vec{A}\;.\;\vec{D}$$

$\vec{X_{rand}}$ represents a randomly selected position vector (a randomly chosen whale) from the current population.

The pseudocode for the Whale Optimization Algorithm (WOA) is outlined in Algorithm 1 [38,41].
**Algorithm 1.** Pseudo-code of the WOAInitialize the whale’s population Xi(i=1,2,…,n)Calculate the fitness of each search agent$X^{*}$
= the best search agent**while** (t < maximum number of iterations)
**for** each search agent

Update a,A,C,landp



**if1** (*p* < 0.5)



**if2** (|A| < 1)




Update the position of the current search agent by the Equation (1)



**else if2** (|A| ≥ 1)




Select a random search agent $\left(X_{rand}\right)$





Update the position of the current search agent by Equation (8)



**end if2**


**else if1** (*p* ≥ 0.5)



Update the position of the current search by the Equation (5)


***end if1***
***end for***
*Check if any search agent goes beyond the search space and amend it*
*Calculate the fitness of each search agent*
*Update* $X^{*}$ *if there is a better solution*
t=t+1 **end while***return* $X^{*}$

#### 3.4.5. Chaotic Whale Optimization Algorithm (CWOA)

The WOA, which is based on the hunting behavior of humpback whales, is a commonly studied algorithm over the past few years due to its simplicity and capability to address challenging optimization problems [38]. However, as with any other metaheuristic technique, WOA is prone to premature convergence and limited diversity within the population and stagnation at the local optimum, particularly when applied to high-dimensional and complex problems such as hyperparameter tuning in deep learning models [60]. To overcome such challenges, ref. [41] suggested CWOA-based solutions where chaotic systems are applied instead, to substitute the primary parameters belonging to WOA that assists in flipping the exploration and exploitation capability of WOA. In an effort to enhance performance, chaos was incorporated in most of the algorithms of meta-heuristic, which leads to a better convergence rate and helps avoid getting trapped in local optima [61]. CWOA is an advanced variant of the WOA, which was introduced to boost the convergence rate and optimization precision of the traditional WOA with chaotic maps. Chaos theory, while being deterministic yet non-predictable, has different exploration capabilities through the avoidance of premature convergence as well as enhancing the algorithm’s capacity to escape from local optima. Various chaotic maps, such as the Logistic map, Tent map, Cubic map, Sine map, Singer map, etc., have been incorporated into the traditional WOA to improve its exploration and exploitation [62]. As stated by [41], the pseudocode for the CWOA is presented in Algorithm 2.
**Algorithm 2.** Pseudo-code of CWOA**Initialize**$\;\mathrm{the}\;\mathrm{generation}\;\mathrm{counter}\;t\;\mathrm{and}\;\mathrm{randomly}\;\mathrm{initialize}\;\mathrm{the}\;\mathrm{whale}\text{’}s\;\mathrm{population}\;X_{i}(i=1,2,\ldots ,n)$**Evaluate** $\mathrm{the}\;\mathrm{fitness}\;\mathrm{of}\;\mathrm{each}\;\mathrm{search}\;\mathrm{agent}\;\mathrm{to}\;\mathrm{find}\;\mathrm{the}\;\mathrm{best}\;\mathrm{search}\;\mathrm{agent}\;X^{*}$**Initialize** $\mathrm{the}\;\mathrm{value}\;\mathrm{of}\;\mathrm{the}\;\mathrm{chaotic}\;\mathrm{map}\;X_{0}$
randomly**while** (t < maximum number of iterations)
**Update** the chaotic number using the respective chaotic map equation

**for** each search agent


**Update** a, A, C, l and p



**if1** (*p* < 0.5)




**if2** (|A| < 1)





**Update** the position of the current search agent by the Equation (1)




**else if2** (|A| ≥
1)





**Select** a random search agent (Xrand)





**Update** the position of the current search agent by Equation (8)




**end if2**



**else if1** (p ≥
0.5)




**Update** the position of the current search by the Equation (5)



**end if1**

**end for**
**Check** if any search agent goes beyond the search space and amend it
**Calculate** the fitness of each search agent
**Update** $X^{*}$ if there is a better solution
t=t+1**end while**return $X^{*}$

#### 3.4.6. Dynamic Chaotic Whale Optimization Algorithm (DCWOA)

The traditional CWOA utilizes a fixed chaotic map throughout the optimization process, which may limit its adaptability in different problem landscapes. The effectiveness of a chaotic approach can also vary based on the optimization landscape, and a single chaotic map may not always provide the best search dynamics. To address this, we propose a Dynamic Chaotic Whale Optimization Algorithm (DCWOA), which dynamically alternates between different chaotic maps, such as logistic and sine map [63] based on the optimization performance. Some other metaheuristic algorithms have also been applied to optimization and feature selection tasks [64,65]. In this study, Chaotic Whale Optimization Algorithm (CWOA) was selected as the base for hyperparameter tuning because of its adoption in solving optimization problems. CWOA has been utilized in several studies due to its ability to enhance the convergence behavior of the original Whale Optimization Algorithm by introducing chaotic dynamics. Refs. [66,67,68,69,70,71,72] have utilized the CWOA method in their respective studies. The objective is to investigate and utilize the impact of chaotic map switching on the effectiveness of the WOA when hyperparameters are tuned in deep learning models.

While traditional methods such as grid search and Bayesian optimization are indeed effective for relatively small or simple hyperparameter spaces, they tend to struggle with high-dimensional and highly non-linear search spaces like the one addressed in our study. Grid search, for instance, becomes computationally prohibitive as dimensionality increases, while Bayesian optimization can be prone to local optima and often lacks the global search capacity needed for complex neural architectures. In contrast, our proposed Dynamic Chaotic Whale Optimization Algorithm (DCWOA) dynamically alternates between different chaotic maps, which enhances its ability to explore the search space broadly and avoid premature convergence. This adaptability and stochastic behavior enable DCWOA to perform robust hyperparameter tuning in deep learning models, offering a level of flexibility and exploratory strength that traditional methods may not easily provide. Additionally, WOA and CWOA, which are the foundation of the DCWOA, have shown promising results in recent studies involving complex optimization scenarios, which further justifies its application in our methodology.

#### 3.4.7. Performance-Based Switching Strategy

The switching mechanism is triggered based on the convergence behavior of the optimization process. A stagnation counter was used to track the number of consecutive iterations where the best fitness (i.e., the loss function in deep learning) remains unchanged. If no improvement is observed for five consecutive iterations, the chaotic map is switched. The updated map governed the chaotic sequence until another stagnation phase was detected. The algorithm alternated between two chaotic maps: logistic map and sine map, depending on the improvement in the best fitness value. Logistic and sine maps were selected through brute-force testing, as they showed the best balance leading to improved convergence in the optimization process. Initially, the logistic map was used as the default chaotic function, defined as in Equation (9):

(9) $$x_{n+1}={rx}_{n}(1-x_{n})$$

where $x_{n}$ represents the chaotic sequence at iteration t, and r is a control parameter, typically set to 4 for full chaos [63]. This map ensures a high degree of randomness and exploration during the initial stages of optimization. To monitor the effectiveness of the search process, DCWOA tracks the number of consecutive iterations without improvement in the best fitness value. If no improvement is observed for five consecutive iterations, the algorithm switches from the logistic map to the sine map, as defined in Equation (10):

(10) $$x_{n+1}=\sin\left(\pi x_{n}\right)$$

If improvement resumes, the algorithm resets the stagnation counter and continues with the currently selected chaotic map. The pseudocode of the CWOA is shown in Algorithm 3.
**Algorithm 3.** Pseudo-code of DCWOA**Initialize**$\;\mathrm{the}\;\mathrm{generation}\;\mathrm{counter}\;t\;\mathrm{and}\;\mathrm{randomly}\;\mathrm{initialize}\;\mathrm{the}\;\mathrm{whale}\text{’}s\;\mathrm{population}\;X_{i}(i=1,2,\ldots ,n)$**Evaluate** $\mathrm{the}\;\mathrm{fitness}\;\mathrm{of}\;\mathrm{each}\;\mathrm{search}\;\mathrm{agent}\;\mathrm{to}\;\mathrm{find}\;\mathrm{the}\;\mathrm{best}\;\mathrm{search}\;\mathrm{agent}\;X^{*}$**Initialize** $\mathrm{the}\;\mathrm{value}\;\mathrm{of}\;\mathrm{the}\;\mathrm{chaotic}\;\mathrm{map}\;X_{0}$
randomly**Set** *no_improvement_count =* 0 and *previous_best_fitness = +*∞
**while** (t < maximum number of iterations)
**Update** the chaotic number using the respective chaotic map equation

**if** no_improvement_count>5


**switch** from the logistic map to the sine map


**Reset** *no_improvement_count =* 0 after switching

**end if**

**for** each search agent 


**Update** a, A, C, l and p based on the chaotic values



**if** (*p* < 0.5)




**if** (|A| < 1)





**Update** the position of the current search agent by the Equation (1)




**else if** (|A| ≥
1)





**Select** a random search agent (Xrand)





**Update** the position of the current search agent by Equation (8)




**end if**



**else if** (p ≥
0.5)




**Update** the position of the current search by the Equation (5)



**end if**

**end for**
**Check** if any search agent goes beyond the search space and amend it
**Calculate** the fitness of each search agent
**If** (leaderfitness≥ *previous_best_fitness*)

**increment** *no_improvement_count* by 1 
**Else**

**set** previous_best_fitness = leader_fitness

**Reset** *no_improvement_count =* 0
**end if**
**Update** $X^{*}$
if there is a better solution
t=t+1**end while**return $X^{*}$

### 3.5. Proposed Custom Learning Rate Scheduler

The learning rate (α) is a critical hyperparameter in deep learning that dictates the step size in weight updates during training. The choice of α significantly impacts the convergence speed and generalization ability of neural networks. In this work, we propose a custom learning rate scheduler that adapts dynamically based on the image size (S) in progressive resizing strategies. The proposed approach ensures smooth adjustments in (α) while maintaining stable training dynamics. The learning rate adjustment is governed by an exponential scaling function that considers an image-dependent factor (β) to modulate in α. The relationship is formulated as Equation (11):

(11) α′=α . β(SS0)

where $\alpha^{\prime }$ is the adjusted learning rate, α is the base learning rate, S represents the image size, and β is the scaling coefficient, defined as Equation (12):

(12) $$\beta =\left\{\begin{array}{cc} 1.0, & S=100\\ 1.05, & S=128\\ 1.1, & S=244\\ 1.15, & S=256\\ 1.2, & S=299 \end{array}\right.$$

Larger image sizes were assigned higher learning rates to accelerate convergence and adapt efficiently to increased feature details. This prevents overfitting at lower resolutions while ensuring stable and efficient training at higher resolutions.

To prevent extreme fluctuations, we impose the following constraints on $\alpha^{\prime }:$ in Equation (13),

(13) $$\alpha^{\prime }=max(\min\left(\alpha^{\prime },\;10\alpha \right),0.1\alpha )$$

where the adjusted learning rate is clamped within an interval of 0.1 α to 10α, ensuring stability across varying image resolutions. The workflow of the proposed custom learning rate is outlined in Algorithm 4.
**Algorithm 4.** Custom learning rate scheduler**Input:** $\mathrm{Base}\;\mathrm{Learning}\;\mathrm{Rate}\;\left(\alpha \right),$ Image Size (S)**Output:** $\mathrm{Adjusted}\;\mathrm{Learning}\;\mathrm{Rate}\;{(\alpha }^{\prime })$**Define** scaling factors β
for different image sizes:
β={100 :1.0, 128 :1.05, 244 :1.1, 256 :1.15, 299 :1.2}**Retrieve** scaling factor for given image size S
:
**If** $S\;\epsilon \;\beta_{\prime }$ **then** $\beta_{S}=\beta [S]$
**Else,** Set $\beta_{S}=1.0$ (default scaling factor)**Compute** the adjusted learning rate:
$\alpha^{\prime }=\alpha \;.\;\beta_{S}^{\left(\frac{S}{S_{0}}\right)}$**Apply** constraint to maintain stability:
$\alpha^{\prime }=max(\min\left(\alpha^{\prime },\;10\alpha \right),0.1\alpha )$**Return** $\alpha^{\prime }$

### 3.6. Proposed Model

**Figure 3:**
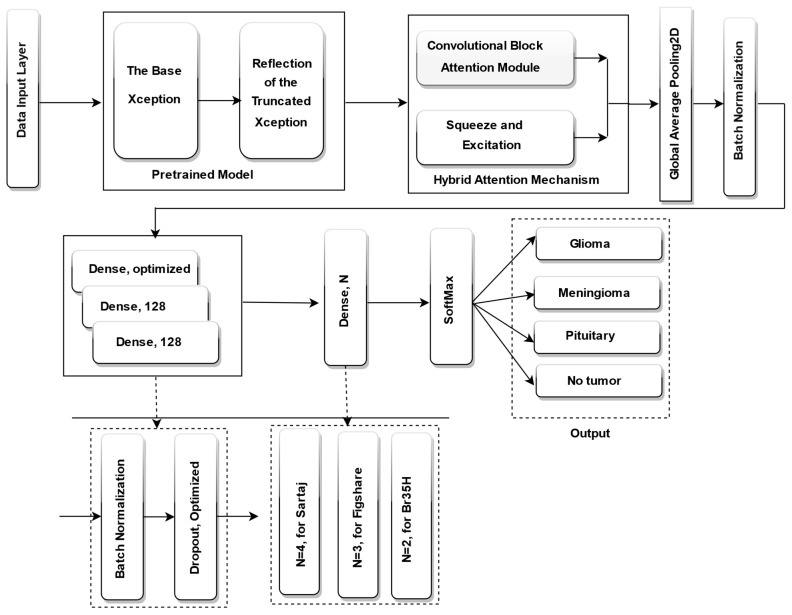
Finetuned Xception pretrained model.

In this study, we suggest an enhanced brain tumor categorization model based on progressive image resizing and the Xception architecture, optimized using the DCWOA. To enhance feature extraction and classification output, we adjust the Xception pretrained model by eliminating its classification layer and integrating a hybrid attention mechanism, which combines squeeze-and-excitation (SE) blocks and CBAM spatial attention. This is followed by global average pooling (GAP) and batch normalization to stabilize training. To enhance feature learning and regularization, the modified architecture incorporated three fully connected (dense) layers, each with 128 neurons, except for the first dense layer, where the number of neurons was dynamically optimized by DCWOA. Each dense layer was followed by batch normalization and dropout. Additionally, DCWOA was employed to optimize key hyperparameters, including the dropout rate, optimizer (Adam, RMSprop, AdamW, Adamax, or Nadam), initial learning rate, and activation function (ReLU, Tanh, or Sigmoid). For classification, a concluding dense layer was incorporated, utilizing a SoftMax activation function for multi-class datasets, including four neurons for the Sartaj dataset and three neurons for the Figshare dataset, using categorical cross-entropy as the loss function. For the BR35H dataset, a binary classification setup was employed, with a single neuron, sigmoid activation, and binary cross-entropy loss function. The fully fine-tuned Xception model is displayed in Figure 3.

**Figure 4:**
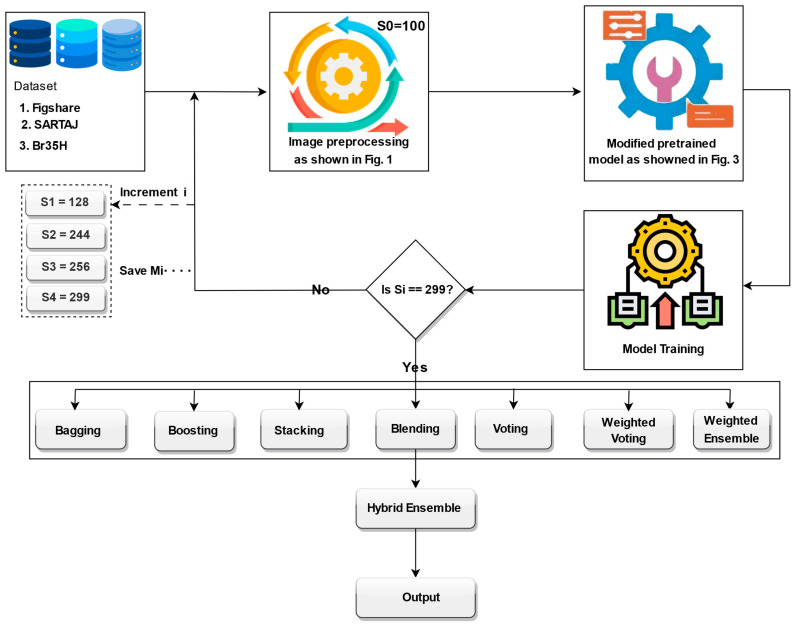
The proposed model.

The training process employed a progressive image resizing method, whereby the model was trained sequentially with progressively larger image sizes: 100, 128, 244, 256, and 299. In each step, the model was trained independently at the given image size, and the weights learned were transferred to the subsequent training step with the subsequent image size. The image sizes were selected based on several practical and methodological considerations. First, we took into account the minimum and maximum input size requirements of the Xception model, which supports input dimensions from 71 × 71 up to 299 × 299. Second, we referenced commonly used image sizes in the recent literature, particularly in brain tumor classification and other medical imaging tasks, where sizes such as 128 × 128, 224 × 224, 256 × 256, and 299 × 299 are frequently employed due to their balance between performance and computational efficiency. Third, we intentionally selected a spread of sizes to support our progressive resizing strategy and to ensure that the ensemble would include models trained at various resolutions, capturing both coarse and fine-grained features. However, other resolutions could also yield competitive results, but our chosen set offers a representative range from low to high resolution. In addition, Xception pretrained layers are unfrozen step by step with a gradually larger image size, allowing the model to learn more and more abstract representations gradually. For promoting efficient convergence, we introduce a learning rate scheduler, which is specially designed to change the learning rate depending on the image size of the input batch, helping the model to learn its training process adaptively. In an attempt to enhance prediction accuracy, generalization, and resilience when trained on different sizes of images, we apply ensemble learning techniques. Specifically, models learned using different image sizes were ensembled using various ensemble strategies, i.e., bagging, boosting, stacking, blending, voting, weighted voting, and weighted ensemble optimized by DCWOA. Finally, the predictions from these ensembles were further hybridized for best classification performance. The complete structure of the proposed model is illustrated in Figure 4. The utilized preprocessing pipeline includes CLAHE for contrast enhancement, bilateral filtering for denoising, and OpenCV cropping to isolate the brain region, which reduces variability caused by artifacts and image borders. Progressive resizing and adaptive training strategies help mitigate differences in resolution. The ensemble approach, especially when optimized using DCWOA, enhances generalization by combining the strengths of diverse models. Each model may handle different types of distortions more effectively, and the dynamic weighting mechanism assigns higher influence to models that perform better under specific conditions. These combined techniques ensure robust performance across heterogeneous datasets.

### 3.7. Performance Evaluation

The effectiveness of the proposed model was evaluated as suggested using four standard classification metrics: recall, F1 score, precision, and accuracy [64,65], as defined in Equations (14)–(17), respectively. These metrics offer a thorough assessment of the model’s performance in accurately classifying brain tumor images.

(14) $$Accuracy=\frac{TP+TN}{TP+TN+FP+FN}$$

(15) $$Precision=\frac{TP}{TP+FP}$$

(16) $$Recall=\frac{TP}{TP+FN}$$

(17) $$F1score=\frac{2*Precision*Recall}{Precision+Recall}$$

## 4. Results

This section presents findings of experimental testing of the proposed method. Comparisons are based on major performance indicators to measure the efficiency of the method. Comparative evaluation with existing methods is also performed to showcase the improvements achieved.

### 4.1. Hyperparameter Optimization Based on DCWOA

In this section, the tuned hyperparameters of various parameters utilized in order to optimize the effectiveness of our deep brain tumor classification model are described. We utilized the DCWOA as an efficient exploration strategy in the hyperparameter space that can identify the best setting by optimizing model generalization and convergence. The DCWOA adaptively adjusted its search behavior by changing between two chaotic maps (logistic map and sine map) when it hit a stagnation point to promote a more diverse and adaptive exploration concerning search space. However, the parameter settings for blending DCWOA with the Xception model are presented in Table 3. Each hyperparameter performs a vital role in enhancing the operation of the Xception model architecture. The optimal iteration count for the optimization algorithm is fixed at 20, while the size of the population, or the total count of potential solutions, is set at 10. The choice of 20 iterations with 10 candidate solutions in the DCWOA-based hyperparameter optimization was made to balance computational efficiency and optimization performance. Given the high training cost of deep models like Xception, a limited search space was necessary. Empirical results showed that the DCWOA reliably converged to stable and effective configurations within these constraints, making further expansion unnecessary. Five hyperparameters are used by the optimization process in simultaneous tuning. The learning rate, which influences weight adjustments during training, is restricted to the interval of 1 × 10^−4^, 1 × 10^−2^. The dropout rate, responsible for network regularization, varies between 0.2 and 0.5. The optimizer, which adjusts network weights to minimize error or loss, is selected from Adam, RMSprop, AdamW, Adamax, and Nadam. The activating function, which presents non-linearity to enable complex pattern learning, is chosen from ReLU, Tanh, and Sigmoid. Additionally, the count of neurons in the initial dense layer, governing the hidden layer’s size, is adjustable between 256 and 1024. To find the total count of training epochs for Xception, multiple values were tested, and 40 epochs were selected as the optimal setting. Table 4 presents the optimal hyperparameter values identified by the DCWOA across the three popular datasets used: Br35H, SARTAJ, and Figshare. This study did not use a fixed random seed. Instead, this study allowed multiple stochastic initializations to observe the stability and robustness of the algorithm across different runs. This variability was crucial in evaluating the sensitivity of DCWOA to randomness and ensuring that the observed performance improvements were not due to a favorable initialization.

### 4.2. Training the Xception Model Using Optimized Hyperparameters

Here, we present the training process of the proposed model, following progressive image resizing. Scripts for all were implemented in Python 3.7.10 on a Jupyter Notebook 6.3.0 hosted on the Kaggle cloud with a disk capacity of 57.6 GiB, 29 GiB of RAM, and a GPU with 16 GiB of memory using the Keras 2.4.3 and TensorFlow 2.4.1 platforms. The optimized Xception model with the optimal hyperparameters set by the DCWOA was utilized as a method for extracting features during training and assessed on the test data for 80 epochs. While the training used all the optimized hyperparameters fixed, the learning rate was updated dynamically after each epoch based on the image size using the proposed custom learning rate scheduler for performance improvement. Batch sizes were adapted based on image size to prevent memory overflow and maintain training speed. Furthermore, the model layers were progressively unfrozen for increasing image size. The adaptive mechanism ensures that task-specific features are reinforced by fine tuning without losing effective pretrained representations. To further enhance performance, the weights of each trained model at a specific image size were saved and used to initialize the succeeding models for the next image size. Categorical cross-entropy was applied to the Figshare and SARTAJ datasets, and binary cross-entropy was applied to the Br35H dataset. The entire process is presented in Algorithm 5. The proposed model was trained independently on each dataset to handle their respective classification tasks. Specifically, the Br35H dataset, being a binary classification problem, was treated separately from the Figshare and SARTAJ datasets, which are multiclass classification problems. Each dataset was preprocessed, trained, and evaluated individually, and the model architecture and hyperparameters were adjusted accordingly to suit the dataset’s structure. This ensures that the results reported for each dataset are valid and reflect the model’s ability to adapt to different classification challenges.
**Algorithm 5.** Training workflow of the proposed model**Input**: Pretrained Xception model, datasets, image sizes S ϵ {100, 128, 244,254,299}**Output:** Predicted Class Label *C***Initialize** pretrained Xception model *M***Remove** classification layer of *M***Integrate** hybrid attention mechanism (SE Block + CBAM Spatial Attention)**Append:**
Global Average Pooling (GAP)
Batch Normalization
Three Dense layers of 128 neurons (except first dense layer, which is optimized)
Batch Normalization (BN) followed by Dropout after each dense layer
Classification layer

Softmax activation with 4 neurons (Sartaj dataset) or 3 neurons (Figshare dataset)

Sigmoid activation with 1 neuron for binary BR3H5 dataset**Initialize** hyperparameter search using DCWOA to optimize:
Count of neurons in the initial dense layer
Dropout rate
Optimizer θ∈{Adam,RMSprop,AdamW,Adamax,Nadam}
$\mathrm{Initial}\;\mathrm{learning}\;\mathrm{rate}\;\propto_{0}$
Activation function f∈{ReLU,Tanh,Sigmoid}**For** each image size S∈{100,128,244,256,299} **do**:
**Set** $\mathrm{learning}\;\mathrm{rate}\;\propto =CustomScheduler(\alpha_{0},\;S)$
**Unfreeze** additional layers of *M* based on *S*
**Train** $\mathrm{model}\;M_{S}$
on dataset resized to S

**Save** $\mathrm{trained}\;\mathrm{weights}\;W_{S}$
**Load** $W_{S}$
into next stage model $M_{S+1}$
**Perform** ensemble learning on models trained at different sizes using:
Bagging, Boosting, Stacking, Blending, Voting, Weighted Voting, Weighted Ensemble with DCWOA**Hybridize** ensemble outputs to obtain final classification model**Classification:**
**Compute** $\mathrm{SoftMax}\;\mathrm{probabilities}\;p=\{p_{1},p_{2},\ldots ..p_{n}\}$ for multi-class datasets.
**Predict** the class label C
as:

C=argmax P
For binary classification, apply a threshold Ƭ
(e.g., 0.5) to the sigmoid output p:


$C=\left\{\begin{array}{c} 1,\;p\geq Ƭ\\ 0,\;p<Ƭ \end{array}\right.$**End** Algorithm

### 4.3. Evaluating the Performance of the Proposed Model

This very section presents a rigorous testing of the effectiveness of the model proposed at various stages with the improvement achieved by using the proposed techniques. The testing is performed on the model trained on various sizes of images and various ensemble techniques such as stacking, bagging, boosting, blending, voting, weighted voting, and a weighted ensemble optimized by the DCWOA. In addition, a hybrid approach that combines all ensemble methods is explored and tested. These performance measures are applied to each of the three datasets used in this study to achieve a comprehensive performance analysis. The model performance was assessed utilizing precision, accuracy, F1 score, recall, and AUC as performance measures.

#### 4.3.1. Model Performance Based on Image Sizes

To assess the impact of image size (resolution) on classification performance, the model was trained and tested on progressively larger image resolutions: 100 × 100, 128 × 128, 244 × 244, 254 × 254, and 299 × 299. This incremental resize strategy traded computation for feature-extraction ability. At lower resolution (100 × 100, 128 × 128), the model processed images more rapidly but less detailed, generating lower accuracy. As image size increased, the model recognized increasingly accurate structural forms, enhancing classification accuracy. The 299 × 299 resolution used the maximum feature representation, leveraging the Xception architecture to the full extent. The performance was in proportion, with the lowest accuracy acquired at 87.91%, 81.60%, and 93.00% for 100 × 100 images, and the highest accuracy acquired was 98.37%, 97.55%, and 98.33% for 299 × 299 images for the three datasets. The results, in Table 5, Table 6 and Table 7, emphasize the impact of image resolution on model performance and validate progressive resizing as a valuable method for improving classification performance.

#### 4.3.2. Model Performance Based on Ensemble Techniques

**Figure 5:**
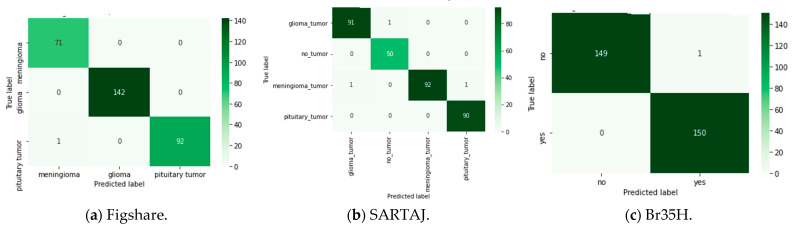
Confusion matrix of the suggested technique based on the three datasets.

**Figure 6:**
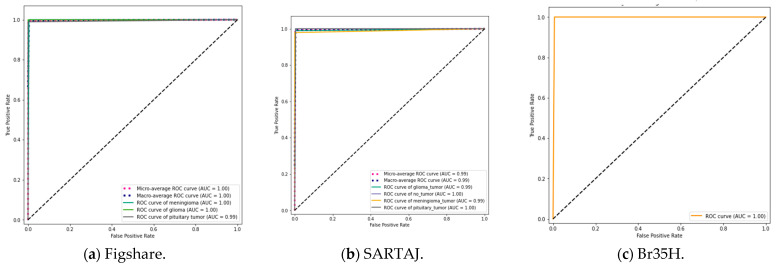
ROC of the suggested technique based on the three datasets.

**Figure 7:**
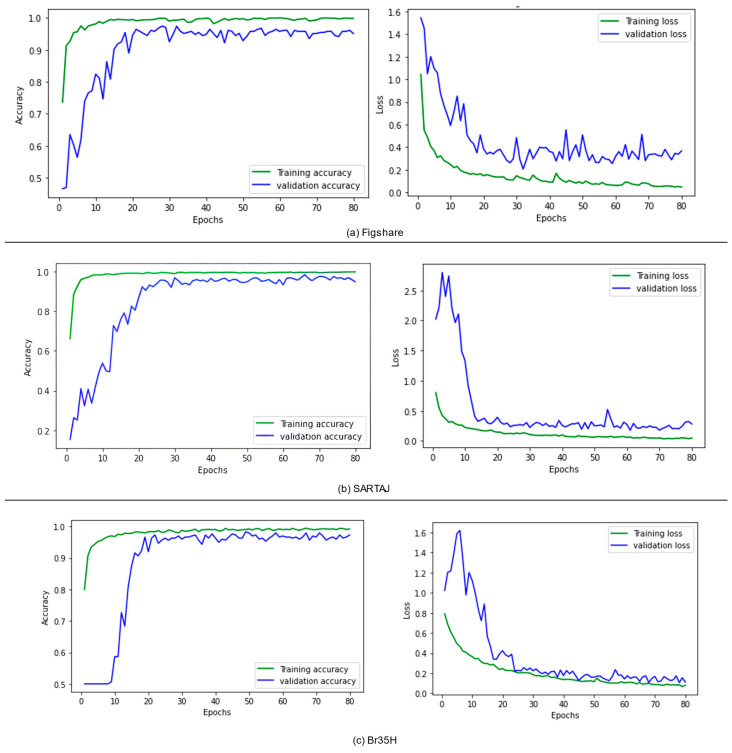
Curves for training and validation accuracy and loss.

To further enhance categorization performance, the suggested model was experimented with different ensemble techniques, including bagging, boosting, stacking, blending, voting, weighted voting, and a weighted ensemble optimized using the DCWOA. All of these methods employ different strategies to increase model stability and predictive capability. Bagging decreases variance by averaging predictions from models trained independently, while boosting gradually enhances weak learners by concentrating on misclassified examples. Stacking and blending stack different models using a meta-learner for the final prediction, while voting aggregates multiple models’ output, with weighted voting assigning higher weightage to higher-performing models. Trained with the DCWOA, the weighted ensemble uses the dynamic weighing of model contributions to classify maximally. On three datasets, performance validated the strengths of these ensemble approaches. The lowest accuracy achieved utilizing the Figshare dataset was 98.04%, while the highest accuracy of 99.67% was reached using the weighted ensemble with the DCWOA. For the SARTAJ dataset, the lowest accuracy was 97.55% using bagging, while boosting had the highest accuracy of 99.09%. For the Br35H dataset, accuracy values were quite high on average, i.e., ranging from 96.33% to 99.67%, with most of the ensemble methods reaching the upper bound. To further assess model reliability, confusion matrices (Figure 5) were utilized to analyze classification errors, unmasking true and false predictions. Figure 5a (Figshare) illustrates performance for three-class tumor classification (glioma, meningioma, and pituitary tumor). The model had great accuracy and correctly classified nearly all cases: 71/71 meningiomas, 142/142 gliomas, and 92/93 pituitary tumors. One pituitary tumor was incorrectly classified as a meningioma, with good model performance and little class confusion. Figure 5b (SARTAJ) shows a four-class classification with both tumor cases and no-tumor cases (glioma tumor, meningioma tumor, pituitary tumor, and no tumor). The model classified almost all cases correctly with minimal misclassifications: one glioma misclassified as no tumor, one meningioma misclassified as glioma, and one pituitary tumor misclassified as meningioma. The no-tumor class showed perfect prediction (50/50), which is essential to reduce false alarms during clinical screening. Figure 5c (Br35H) illustrates a binary distinction between no-tumor and tumor samples. The model was nearly perfect in terms of accuracy, correctly classifying 149/150 no-tumor images and 150/150 tumor images. Only one false positive was reported. The confusion matrices in total confirm that the model generalizes extremely well for different tasks of classification, with immense diagnostic potential, particularly in minimizing false negatives, which is of utmost importance in medical applications. In addition, the ROC curves (Figure 6) reveal the discriminative capacity of the model on datasets and accuracy along with the loss curves. Figure 7a–c show training stability and convergence trends. The results, as summarized in Table 8, Table 9 and Table 10, reflect the impact of ensemble learning and optimization in enhancing classification outcomes for Figshare, Br35H, and SARTAJ datasets, respectively. However, the hybrid approach that combines all ensemble techniques did not yield any further improvement in accuracy. The highest classification performance was achieved using individual ensemble methods for all three datasets, indicating that certain ensemble strategies, such as a weighted ensemble optimized by the DCWOA and boosting, were more effective in enhancing model accuracy than a generalized hybrid combination.

Our proposed classification model, like many deep learning systems, could be vulnerable to several security threats, particularly adversarial and poisoning attacks. For instance, attackers may manipulate input data or training samples in subtle ways to alter the model’s predictions without detection, a concern thoroughly explored in the work by [73], which demonstrates how even small perturbations can compromise diagnostic models. Additionally, as healthcare increasingly integrates AI into Internet-of-Things (IoT) frameworks, concerns around data integrity, authentication, and transmission security also emerge. Refs. [74,75] in studies highlight the risks of model inversion, evasion attacks, and unauthorized data access, especially in resource-constrained or remote environments. Moreover, ref. [76] underscores the need for secure and efficient AI deployment in medical monitoring systems, especially where models may be exposed to edge-based or cloud-based threats. While our current work focuses primarily on model accuracy and interpretability, we acknowledge that future extensions should include robust defense mechanisms such as adversarial training, input verification, and secure model deployment strategies to guard against these emerging security threats.

### 4.4. Comparison of the Proposed Model with Other State-of-the-Art Models in the Literature

In this section, we provide a comparative analysis of our model alongside recently developed state-of-the-art approaches. As evident from Table 11, the proposed model achieved the highest accuracy of 99.67%, which is better compared to most existing methods. The papers were selected based on their relevance to our problem domain, as they address similar challenges and their reflection of recent advancements in the field. Additionally, they use the same dataset as our study, ensuring a consistent and fair comparison of model performance.

### 4.5. Addressing Data Imbalance

There is a presence of imbalance in class distribution, which can indeed influence model training and evaluation if not properly addressed. To mitigate the impact of this imbalance, we applied data augmentation to the SARTAJ and Br35H datasets, which helped artificially expand the minority classes and reduce potential bias. Although augmentation was not applied to the Figshare dataset due to earlier experimentation showing no improvement, we employed ensemble techniques to all three datasets, which are known to be robust against class imbalance by combining diverse learners and correcting biases of individual models through averaging or majority voting. Importantly, we did not rely solely on accuracy as a performance metric. Instead, we reported Precision, Recall, and F1 score, which are more informative and widely accepted in the literature for evaluating models trained on imbalanced datasets. These metrics allowed us to assess performance across all classes, including minority ones.

### 4.6. Model Evaluation and Interpretability Using Grad-CAM

**Figure 8:**
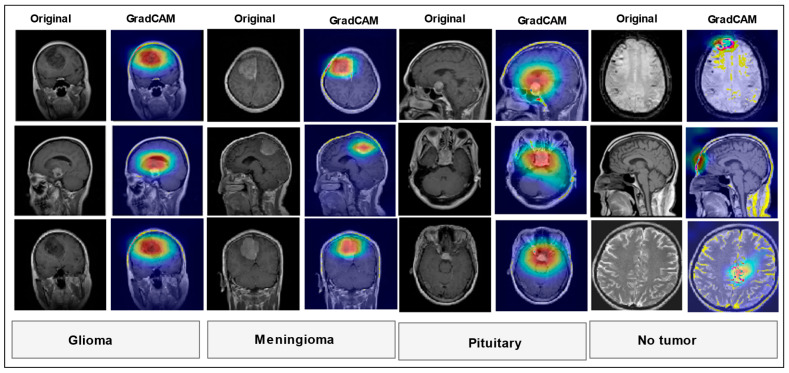
Crad-CAM visualization.

To assess the internal decision making of our proposed deep learning model and increase its clinical explainability, we utilized Gradient-weighted Class Activation Mapping (Grad-CAM). Grad-CAM is a common visualization tool that produces class-discriminative heatmaps, enabling the detection of spatial areas within the input image that contribute most significantly to the predictions of the model. In our study, we applied Grad-CAM to both tumor-positive and tumor-negative brain MRI slices to assess whether the model focuses on clinically relevant anatomical structures during classification. For tumor-positive cases, the Grad-CAM heatmaps consistently highlighted regions corresponding to tumor masses. These areas aligned well with known tumor locations by comparing the original images and GradCAM images. For tumor-negative cases, the heatmaps displayed minimal and diffuse activation, with no strong localized focus. This suggests that the model does not falsely fixate on normal structures when no tumor is present, which is indicative of a low false-positive tendency. An example visualization is shown in Figure 8, where the left panel depicts the original MRI image and the right panel overlays the Grad-CAM heatmap. Bright regions in the heatmap (red/yellow) indicate high model attention, clearly corresponding to the tumor area in positive cases. Such interpretability is crucial for building trust in AI-assisted diagnostics, especially in sensitive applications such as neuro-oncology.

## 5. Conclusions

This paper suggested a more advanced brain tumor identification model using progressive image resizing, a hybrid attention-based architecture of modified Xception, and an optimized ensemble learning approach. Through progressively improving image resolution in training, the model effectively extracts both high-level and low-level features with improved classification accuracy. The addition of Squeeze-and-Excitation (SE) blocks and CBAM spatial attention enhances feature extraction, while the DCWOA optimizes hyperparameters for better generalizability. Experimental findings reveal that higher image resolution is accountable for enhanced classification accuracy, where peak performance is achieved at 299 × 299 resolution. Ensemble learning approaches also indicate a significant enhancement of model resilience with the top-performing model obtained with the DCWOA-optimized weighted ensemble. The weighted ensemble optimized using the DCWOA outperformed other ensemble methods in the Figshare dataset, achieving up to 99.67% accuracy. It dynamically assigns weights to individual models based on performance. Boosting also outperformed other ensemble methods, particularly on the SARTAJ dataset. Hybrid combinations provided marginal improvements or redundancy, highlighting that performance gains depend on model diversity and dataset characteristics. This research substantiates the effectiveness of the introduced approach towards the enhanced automated detection of brain tumors. This proposed approach improves the precision of brain tumor classification, thereby facilitating more accurate automated diagnosis. Progressive resizing, attention mechanisms, and ensemble learning enhance feature extraction and model stability. Hyperparameter optimization with the DCWOA ensures improved generalization, positioning the model more suitable for real-world medical applications. These findings provide a basis for future AI-powered advancements in medical imaging and clinical decision support. Despite high accuracy, the model was trained and tested on publicly available datasets, which may not reflect real-world clinical variability. Potential risks include overfitting, data distribution shift, and reliance on biased datasets. From an ethical perspective, decisions made by AI systems must be transparent, explainable, and overseen by clinical experts. In clinical settings, our model should serve as a decision-support tool, not a replacement for human judgment.

## 6. Future Works

The future works can look at the integration of transformer-based model architectures, such as Vision Transformers (ViTs), to enhance feature representation and improve classification efficiency. An investigation of other pretrained models can be looked into, with the possibility of complementary feature extraction capabilities, further enhancing the model’s robustness. Future work will also investigate case-specific error patterns, including the visual inspection of false positives/negatives, and assess their clinical implications to better understand model limitations and areas for improvement

## Figures and Tables

**Table 1:** Data augmentation strategy.

Number	Augmentation Methodology	Parameters
1.	Rotation	20
2.	Width shift	0.2
3.	Height shift	0.2
4.	Shear	0.2
5.	Zoom	0.2
6.	Bright	[0.2, 1.0]
7.	Horizontal flip	True

**Table 2:** Preprocessing evaluation.

Stage	Metric	Value	Interpretation
Original Image	Entropy	6.936	High texture complexity, rich information content
	Mean Intensity	62.268	Balanced brightness level
After Cropping	Entropy	6.942	Slight increase in detail, cropping preserved or enhanced local complexity
	Mean Intensity	62.268	No change, cropped region maintains original intensity distribution
After CLAHE	PSNR	18.226	Improved pixel-level similarity with better contrast
	SSIM	0.738	Strong structural preservation despite contrast enhancement
After Bilateral	PSNR	18.399	Slightly better than CLAHE, effective edge-preserving denoising
	SSIM	0.672	Slight structural distortion compared to CLAHE, but still acceptable
	SNR	8.233	High signal-to-noise ratio, excellent noise reduction while retaining details

**Table 3:** Parameter configurations for using DCWOA with Xception and the corresponding dataset.

Parameter	Value
Highest iteration limit	20
Population size	10
Dimension	5
Range of learning rate	$[{1\times 10}^{-4},\;{\;1\times 10}^{-2}$ ]
Optimizer	[Adam, RMSprop, AdamW, Adamax, Nadam]
Activation function	[ReLU, Tanh, Sigmoid]
Rate of dropout	[0.2, 0.5]
Range of number of neurons	[254, 1024]
Maximum training epochs of Xception	40
Chaotic map	[logistic, sine]

**Table 4:** Optimal hyperparameter values determined using DCWOA.

Hyperparameter	Optimal Value		
	Figshare	SARTAJ	Br35H
Learning rate	0.001	0.001	0.001
Optimizer	Nadam	Nadam	Nadam
Activation function	Sigmoid	Relu	Sigmoid
Rate of dropout	0.5	0.5	0.5
Number of neurons in the first layer	1024	1024	1024

**Table 5:** Evaluation results based on the Figshare dataset for each of the image resolutions.

Image	Accuracy	F1 Score	Recall	Precision	AUC
100 × 100	87.91	86.97	87.12	87.13	91
128 × 128	92.48	91.89	92.45	91.52	94
244 × 244	95.42	95.06	9587	9458	97
254 × 254	98.04	97.76	97.76	97.76	99
299 × 299	98.37	98.12	98.33	97.99	99

**Table 6:** Evaluation results based on the SARTAJ dataset for each of the image resolutions.

Image	Accuracy	F1 Score	Recall	Precision	AUC
100 × 100	81.60	81.64	83.31	81.08	88
128 × 128	86.20	86.68	86.47	88.15	91
244 × 244	94.79	94.62	95.21	94.24	97
254 × 254	96.93	97.16	97.10	97.37	98
299 × 299	97.55	97.49	97.60	97.42	98

**Table 7:** Evaluation results based on the Br35H dataset for each of the image resolutions.

Image	Accuracy	F1 Score	Recall	Precision	AUC
100 × 100	93.00	93.00	93.00	93.16	93
128 × 128	93.67	93.67	93.67	93.67	94
244 × 244	96.67	96.67	96.67	96.67	97
254 × 254	96.67	96.67	96.67	96.70	97
299 × 299	98.33	98.33	98.33	98.33	98

**Table 8:** Evaluation results based on the Figshare dataset for each of the image ensembles.

Ensemble	Accuracy	F1 Score	Recall	Precision	AUC
Bagging	98.04	97.80	97.54	98.11	99
Boosting	99.02	98.89	98.84	98.94	99
Stacking	98.37	98.15	97.81	98.58	99
Blending	98.37	98.15	97.81	98.58	99
Voting	98.04	97.80	97.54	98.11	99
Weighted Voting	98.37	98.15	97.81	98.58	99
Weighted Ensemble with DCWOA	99.67	99.59	99.54	99.64	1.00
Voting + Boosting Ensemble	98.04	97.80	97.54	98.11	99
Weighted Voting + Blending	98.37	98.15	978.01	98.58	99
Voting + Stacking + Weighted Ens.	98.04	97.80	97.54	98.11	99
Voting + Stacking + Weighted Ens.	98.69	98.49	98.22	98.81	99
Hybrid	99.02	98.83	98.65	99.05	

**Table 9:** Evaluation results based on the Br35H dataset for each of the image ensembles.

Ensemble	Accuracy	F1 Score	Recall	Precision	AUC
Bagging	99.67	99.67	99.67	99.67	1
Boosting	96.33	96.33	96.58	96.33	96
Stacking	99.67	99.67	99.67	99.67	1
Blending	99.67	99.67	99.67	99.67	1
Voting	99.67	99.67	99.67	99.67	1
Weighted Voting	99.67	99.67	99.67	99.67	1
Weighted Ensemble with DCWOA	99.67	99.67	99.67	99.67	1
Voting + Boosting Ensemble	99.67	99.67	99.67	99.67	1
Weighted Voting + Blending	99.67	99.67	99.67	99.67	1
Voting + Stacking + Weighted Ens.	99.67	99.67	99.67	99.67	1
Voting + Stacking + Weighted Ens.	99.67	99.67	99.67	99.67	1
Hybrid	99.67	99.67	99.67	99.67	1

**Table 10:** Evaluation results based on the SARTAJ dataset for each of the image ensembles.

Ensemble	Accuracy	F1 Score	Recall	Precision	AUC
Bagging	97.55	97.49	97.40	97.61	98
Boosting	99.09	99.07	98.96	99.20	99
Stacking	98.77	98.69	98.68	98.70	99
Blending	98.77	98.69	98.68	98.70	99
Voting	98.77	98.69	98.69	98.70	99
Weighted Voting	98.77	98.69	98.68	98.70	99
Weighted Ensemble with DCWOA	98.77	98.80	98.69	98.92	99
Voting + Boosting Ensemble	98.77	98.69	98.69	98.70	99
Weighted Voting + Blending	98.77	98.69	98.68	98.70	99
Voting + Stacking + Weighted Ens.	98.77	98.69	98.69	98.70	99
Voting + Stacking + Weighted Ens.	98.77	9869	98.68	98.70	99
Hybrid	98.77	98.69	98.68	98.70	99

**Table 11:** Comparative analysis.

Reference	Dataset	Accuracy
[5]	Figshare	97.80
[44]	Figshare	96.00
[45]	Figshare, Br35H	96.51, 4.91
[47]	Figshare	95.82
[50]	Figshare	97.77
[51]	Combined (Figshare, SARTAJ, Br35H)	98.00
[52]	Figshare, SARTAJ, Br35H	98.13
[53]	Figshare	94.0
[54]	Figshare	97.00
Proposed model	Figshare	99.67
Proposed model	SARTAJ	99.09
Proposed model	Br35H	99.67

## Data Availability

The original contributions presented in the study are included in the article, further inquiries can be directed to the corresponding author.
